# Updated recommendations from the Canadian National Consensus Meeting on HER2/*neu* testing in breast cancer

**DOI:** 10.3747/co.2007.131

**Published:** 2007-08

**Authors:** W. Hanna, F.P. O’Malley, P. Barnes, R. Berendt, L. Gaboury, A. Magliocco, N. Pettigrew, S. Robertson, S. Sengupta, B. Têtu, T. Thomson

**Keywords:** her2/*neu*, breast cancer, testing guidelines, immuno-histochemistry, fluorescence in situ hybridization, quality assurance, quality control

## Abstract

Testing for her2/*neu* in breast cancer at the time of primary diagnosis is now the standard of care. Accurate and standardized testing methods are of prime importance to ensure the proper classification of the patient’s her2/*neu* status. A meeting of pathologists from across Canada was convened to update the Canadian her2/*neu* testing guidelines. This National her2/*neu* Testing Committee reviewed the recently published American Society of Clinical Oncology/ College of American Pathologists (asco/cap) guidelines for her2/*neu* testing in breast cancer. The updated Canadian her2/*neu* testing guidelines are based primarily on the asco/cap guidelines, with some modifications. It is anticipated that widespread adoption of these guidelines will further improve the accuracy of her2/*neu* testing in Canada.

## 1. INTRODUCTION

The human epidermal growth factor 2 (her2/*neu*) gene is amplified in approximately 18%–20% of breast cancers 1–4, and it is the primary mechanism for her2 protein overexpression 5. Overexpression of her2 is predictive of response to particular therapies, including trastuzumab (Herceptin: Genentech, San Francisco, California, U.S.A.) treatment in the meta-static and adjuvant settings 1,6–10.

Recognizing the importance of accurate her2/*neu* status assessment, pathologists from across Canada gathered to share local testing experiences and insights. The meeting was chaired by Dr. Wedad Hanna and Dr. Frances P. O’Malley, who facilitated the collaboration to create a Canadian consensus statement for her2 testing procedures.

**FIGURE 1:**
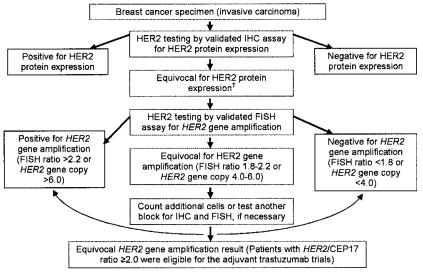
Algorithm for immunohistochemistry (ihc) and fluorescence *in situ* hybridization (fish). Based partly on Wolff *et al.*^1 †^ Evaluation of equivocal ihc results includes retesting with fish or other validation brightfield *in situ* hybridization techniques [chromogenic in situ hybridization (cish) or silver-enhanced *in situ* hybridization (sish)].

The American Society of Clinical Oncology (asco) and the College of American Pathologists (cap) convened an expert panel to develop a U.S. guideline to improve the accuracy of her2 testing in invasive breast cancer. This asco/cap guideline was reviewed by the panel of Canadian pathologists. The data from that review and the experience of the panel members were used to create the current update of the Canadian her2 testing guidelines and algorithm (Figure 1). The updated information is presented here.

## 2. CONSENSUS PARTICIPANTS

### Co-chairs

Wedad Hanna md (Toronto, Ontario) and Frances P. O’Malley md (Toronto, Ontario)

### Delegates

Penelope Barnes md (Halifax, Nova Scotia), Richard Berendt md (Edmonton, Alberta), Louis Gaboury md (Montreal, Quebec), Anthony Magliocco md (Calgary, Alberta), Norman Pettigrew md (Winnipeg, Manitoba), Susan Robertson md (Ottawa, Ontario), Sandip Sengupta md (Kingston, On-tario), Bernard Têtu md (Québec City, Quebec), and Thomas Thomson md (Vancouver, British Columbia)

The information that follows is based both on the experience of the consensus participants and on the recently published asco/cap Guideline Recommendations for her2/*neu* Testing in Breast Cancer.

## 3. ISSUES CONSIDERED BY THE CONSENSUS PARTICIPANTS

### 3.1 Testing at Diagnosis

The her2/*neu* gene has proved to be a significant prognostic and predictive biologic marker in breast cancer 1. Thus, the current standard of care is to test all patients with invasive breast cancer for her2/*neu* at the time of diagnosis 1.

The panel agreed that standardized and validated tests should be performed. Available data do not show superiority for either immunohistochemistry (ihc) or *in situ* hybridization (ish) as a predictor of benefit from Herceptin therapy. The testing algorithm proposed for all invasive breast cancers is based on the very high concordance between her2/*neu* gene amplification and protein expression as determined using accurate and reproducible assay methods. Thus, the Canadian consensus agrees with the asco/cap guidelines and is based on testing first with ihc and then retesting equivocal cases using ish [either fluorescent (fish) or another validated brightfield ish method such (as chromogenic cish) or silver-enhanced (sish)] 1. Throughout the current document, the use of fish methodology is not exclusive; labs can use other validated ish methods to assess equivocal cases both for clinical case and quality assurance (qa) activities.

The guidelines and variables related to accurate testing are discussed under the headings pre-analytic, analytic, and post-analytic.

### 3.2 Pre-analytic

#### 3.2.1 Tissue Handling and Fixation

Time from specimen excision to placement in fixative should be minimized. Samples should be sliced immediately at 5–10 mm intervals after appropriate gross inspection and designation of margins and then placed in a sufficient volume of 10% neutral buffered formalin 1. Optimal time of fixation is 24–48 hours in 10% neutral buffered formalin, but a period of at least 6 hours 11 is sufficient for core biopsy specimens. A fixation time longer than 48 hours for lumpectomy/ mastectomy specimens is not an exclusion criterion for her2 testing (Table I) provided that the specimen has been appropriately sectioned to allow adequate fixation as described above. Under-fixation is more critical than over-fixation; less than 6 hours’ fixation time precludes the specimen from being used for her2 testing. The fixation requirements described above pertain to testing both with ihc and with fish.

### 3.3 Analytic

#### 3.3.1 Assay validation

When validating a new antibody, 25–100 samples should be tested 1. However, if the lab has little experience with performing her2 testing, a minimum of 100 sample tests is advisable. An assay accuracy of 95% concordance rate should be achieved for both the positive and negative categories. Adequate validation should be ensured, preferably by using 50% cases that are unequivocally positive and 50% cases that are unequivocally negative. Validation documentation must be kept. Any modification to the procedure requires additional validation to ensure accurate performance.

#### 3.3.2 Type of Antigen Retrieval

Stringent compliance to validated standard operating procedures developed in assay validation must be adhered to and quality control (qc) documentation must be in place.

#### 3.3.3 Use of Standard Control Materials

The controls should include positive (overexpressed/ amplified) and negative (not overexpressed/not amplified) cases, plus a low amplified/low protein over-expression case if possible. The control tissue should be fixed and processed in the same manner as the patient samples.

#### 3.3.4 Use of Automated Lab Methods

The use of correctly operated automated staining protocols and equipment are acceptable, but validated methods must be used. Maintenance and service records should be regularly updated and filed in the laboratory.

### 3.4 Post-analytic

#### 3.4.1 Image Analysis

Use of image analysis systems can be useful to enhance reproducibility of scoring; pathologists must supervise all image analyses.

#### 3.4.2 Mandatory reporting elements, IHC

The panel agreed that mandatory reporting elements testing include these items for ihc 1:

- Patient identification information
- Physician identification
- Date of service
- Specimen identification (case and block number)
- Specimen site and type
- Specimen fixative type (if not 10% neutral buffered formalin)
- Time to fixation (if available)
- Duration of fixation (if available)
- Antibody clone and vendor
- Method used (test and vendor)
- Image analysis method (if used)
- Adequate controls
- Adequacy of sample for evaluation
- Results:
  - Percentage of invasive tumour cells exhibiting complete membrane staining
  - Uniformity of staining: present or absent
  - Homogenous, dark circumferential pattern: present or absent
- Interpretation (Table II):
  - Positive (for her2 protein expression)
  - Equivocal (fish will be done and reported)
  - Negative (for her2 protein expression)
  - Not interpretable

#### 3.4.3 Mandatory reporting elements, FISH

The panel agreed that mandatory reporting elements for fish testing include these items^1^:

- Patient identification information
- Physician identification
- Date of service
- Specimen identification (case and block number)
- Specimen site and type
- Specimen fixative type (if not 10% neutral buffered formalin)
- Time to fixation (if available)
- Duration of fixation (if available)
- Identification of probe (or probes)
- Method used (specifics of test and vendor)
- Image analysis method
- Adequate controls
- Adequacy of sample for evaluation (adequate number of invasive tumour cells present)
- Results:
  - Number of invasive tumour cells counted
  - Number of observers (optional)
  - Average number of her2 signals per nucleus or tile^a^
  - Average number of chromosome enumeration probe 17 (cep 17) signals per nucleus or tile^a^
  - Ratio of average her2 signals to cep 17 signals
- Interpretation (Table III):
  - Positive (amplified)
  - Equivocal
  - Negative (not amplified)
  - Not interpretable
  - If ihc is being done because of problems with assay or results, that fact should also be included

#### 3.4.4 Volume

Per the United Kingdom guidelines, each lab should tests perform at least 250 ihc 12. If the lab also performs fish, then at least 100 fish tests should be performed annually. However, given the complexity of the procedure and the experience needed, it is advisable and preferable to perform at least 200 fish tests annually.

Appropriate training for pathologists should take place, and the number of tests performed by each pathologist should be considered to ensure competency. Test volume should be assessed in conjunction with the lab’s adherence to strict qc and qa practices. Technologists should undergo appropriate training, including ongoing education in fish technology and interpretation.

### 3.5 QA Procedures

#### 3.5.1 Optimal Internal QA Procedures

The panel recommends that initial test validation should take place together with ongoing qc and equipment maintenance. Initial and ongoing education of laboratory personnel, training, and competency assessment should also be implemented. The use of standardized operating procedures, including routine use of control materials, should be enforced, and modified procedures should be revalidated. Finally, ongoing competency assessment and education of pathologists should take place.

#### 3.5.2 Optimal External Proficiency Assessment

The panel agreed that participation in an external proficiency testing program with at least two testing events (mailings) annually is mandatory. Also, satisfactory performance requires at least 90% correct responses in graded challenges for either test. Unsatisfactory performance will require a laboratory to respond according to accreditation agency program requirements.

#### 3.5.2 Optimal Laboratory Accreditation

Onsite inspection should take place every other year with an annual requirement for self-inspection. Review of laboratory validation, procedures, qa results and processes, results, and reports should be put into place. Unsatisfactory performance results in suspension of laboratory testing for her2 for that method.

#### 3.5.3 Statistical Requirements for Assay Validation

“Sensitivity” is defined as the percentage of positive test results obtained when evaluating only specimens that are truly positive. “Specificity” is defined as the percentage of negative test results reported when only truly negative specimens are evaluated. “Overall accuracy” (concordance) combines sensitivity and specificity into a single measure of the percentage of cases (positive and negative) for which the assay result agrees with the true status. Note that overall accuracy (concordance rate) can be strongly influenced by the positive–negative mix of the test case set if the sensitivity and specificity rates are not similar.

Examples of external qa programs include these:

- cap (www.cap.org)
- U.K. National External Quality Assessment Service (neqas: www.ukneqas.org.uk)
- Nordic Immunohistochemical Quality Control (NordiQC: www.nordiqc.org)
- Ontario [overseen by Quality Management Program–Laboratory Services (www.qmpls.org) and Cancer Care Ontario (www.cancercare.on.ca)]

#### 3.5.4 Algorithm for IHC and FISH

The panel agreed that pathologists should test at diagnosis, starting with ihc and then, in equivocal cases, moving to fish or to another validated brightfield *in situ* hybridization method such as cish or sish. In cases in which the available sample is small or the sample is a core biopsy in the neoadjuvant setting, it is preferable to perform ish as the initial test (Figure 1).

## 4. CONCLUSIONS

Considering the prognostic and predictive significance of determining her2/*neu* status in invasive breast cancer, her2/*neu* testing performed at the time of initial diagnosis is now the standard of care. In line with the updated asco/cap guidelines, the Canadian consensus guidelines provide recommendations on how to evaluate and report her2/*neu* over-expression and gene amplification. In summary, the guidelines outline specific criteria that laboratories must meet before engaging in her2/*neu* testing.

The 2007 her2/*neu* testing algorithm integrates both the ihc and the fish algorithms as published in the asco/cap guidelines. It is important to note that the cut-offs for positivity for both ihc and fish have been updated; a positive ihc result is now defined as >30% strong complete membrane staining; a positive fish result is defined as a ratio > 2.2 or a *her2* gene copy > 6.0.

With continual efforts being made to optimize her2/*neu* testing accuracy, the testing guidelines and algorithm will be regularly updated to convey current practices.

## Figures and Tables

**TABLE I:** Exclusion criteria, immunohistochemistry (IHC) and fluorescence *in situ* hybridization (FISH)

Sample exclusion criteria to perform or interpret a her2 ihc assay	Sample exclusion criteria to perform or interpret a her2 fish assay
Tissue fixed using other than 10% neutral buffered formalin a Excisional and needle biopsies fixed for less than 6 hours	Samples with only limited invasive cancer difficult to define under ultraviolet light
Core needle biopsies with	Tissue fixed in fixatives other than 10% neutral buffered formalin a
• edge or retraction artifact involving entire core	Excisional or core biopsies fixed in formalin for less than 6 hours
• crush artifact (thin-gauge vacuum-extraction needle samples)	Controls with unexpected results
Tissues with strong membrane staining of internal normal ducts or lobules	fish signals non-uniform (<75% identifiable) Background obscures signal (>10% of signals over cytoplasm)
Tissues where controls exhibit unexpected results	Non-optimal enzymatic digestion (poor nucleus resolution, persistent autofluorescence)

a If a laboratory uses fixatives other than buffered formalin, it must validate the performance characteristics of the assay to show that those characteristics are concordant with results using buffered formalin in the same samples.

**TABLE II:** Interpretation criteria, immunohistochemistry (IHC)

Result category	ihc score (her2 protein expression)	Interpretation criteria
Positive	3+	Strong, complete, homogeneous membrane staining (chicken-wire pattern) in >30% of cells
Equivocal^a^	2+	Strong, complete membrane staining (chicken-wire pattern) in ≤30% of cells Weak or moderate heterogeneous complete membrane staining in at least
Negative	0–1+	10% of cells No staining (0), or weak, incomplete membrane staining (1+) in any percentage of cells

a Confirm by fluorescence *in situ* hybridization (fish) analysis of

original sample.

**TABLE III:** Interpretation criteria, fluorescence *in situ* hybridization (FISH)

Result category	fish score (her2 gene amplification)
Positive	her2:cep 17 ratio > 2.2 or Average her 2 gene copy number > 6 a
Equivocal	her2: cep 17 ratio = 1.8–2.2^b^,^c^ or Average her 2 gene copy number = 4–6 a
Negative	her2:cep 17 ratio < 1.8 or Average her 2 gene copy number < 4 a

a Signals or nucleus for test systems without an internal chromosome 17 centromeric probe.

b Count additional cells, or test another block for immunohistochemistry (ihc) and fish if necessary.

c Patients with a her2 gene amplification of ≥2 were eligible for adjuvant trastuzumab trials.

cep 17 = chromosome enumeration probe 17.

## Footnotes

a “Tile” is the unit used for image-system counting.
